# The TCA Pathway is an Important Player in the Regulatory Network Governing *Vibrio alginolyticus* Adhesion Under Adversity

**DOI:** 10.3389/fmicb.2016.00040

**Published:** 2016-02-02

**Authors:** Lixing Huang, Li Huang, Qingpi Yan, Yingxue Qin, Ying Ma, Mao Lin, Xiaojin Xu, Jiang Zheng

**Affiliations:** Key Laboratory of Healthy Mariculture for the East China Sea, Ministry of Agriculture, Fisheries College, Jimei UniversityXiamen, China

**Keywords:** *Vibrio alginolyticus*, adhesion, TCA pathway, environmental stresses

## Abstract

Adhesion is a critical step in the initial stage of *Vibrio alginolyticus* infection; therefore, it is important to understand the underlying mechanisms governing the adhesion of *V. alginolyticus* and determine if environmental factors have any effect. A greater understanding of this process may assist in developing preventive measures for reducing infection. In our previous research, we presented the first RNA-seq data from *V. alginolyticus* cultured under stress conditions that resulted in reduced adhesion. Based on the RNA-seq data, we found that the Tricarboxylic acid cycle (TCA pathway) might be closely related to adhesion. Environmental interactions with the TCA pathway might alter adhesion. To validate this, bioinformatics analysis, quantitative Real-Time PCR (qPCR), RNAi, and *in vitro* adhesion assays were performed, while *V. alginolyticus* was treated with various stresses including temperature, pH, salinity, and starvation. The expression of genes involved in the TCA pathway was confirmed by qPCR, which reinforced the reliability of the sequencing data. Silencing of these genes was capable of reducing the adhesion ability of *V. alginolyticus*. Adhesion of *V. alginolyticus* is influenced substantially by environmental factors and the TCA pathway is sensitive to some environmental stresses, especially changes in pH and starvation. Our results indicated that (1) the TCA pathway plays a key role in *V. alginolyticus* adhesion: (2) the TCA pathway is sensitive to environmental stresses.

## Introduction

*Vibrio alginolyticus* is an important opportunistic pathogen for marine organisms (Patterson et al., 1988). *V. alginolyticus* is an ubiquitous organism in seawater and has been associated with several epidemics of vibriosis in cultivated marine animals, including fish, shellfish, crustaceans (Wang et al., 2012), and coral reefs (Xie et al., 2013). It has also been reported to cause otitis and wound infections in human beings (Kong et al., 2015).

Large yellow croaker (*Pseudosciaena crocea*) is an economically important cultivated marine species in China. *V. alginolyticus* is the main pathogenic bacterium of the cultured large yellow croaker, which has led to considerable economic losses (Yan et al., 2007; Huang et al., 2008). As previously reported, the temperature (Yeh et al., 2010), salinity (López-Hernández et al., 2015), pH (Li and Chen, 2008), and starvation (Yi et al., 2008) are the primary environmental factors of epidemic vibriosis in aquaculture.

The pathogenic process of bacteria is generally divided into adhesion, invasion, colonization, proliferation and production of toxins (Huang et al., 2015a). The ability to adhere to mucus is a crucial bacterial virulence mechanism. Bacterial adhesion is a complex process related to bacterial factors and environmental factors (Yan et al., 2007). In recent years, studies have shown that the temperature, salinity, pH, starvation and heavy metal could affect the growth and adhesion ability of *Vibrio* species. López-Hernández et al. (2015) found that vibriosis is more likely to occur during spring and summer, which offer appropriate temperature. pH, an important environmental factor, has a substantial influence on bacterial attachment. Balebona et al. (1995) found optimum adhesion of *Vibrio* strains to the skin mucus of *Sparus aurata* at pH 8.1. Metals were introduced into the environment from various sources rapidly in the last century (Xiao et al., 2015). Metals can affect the biochemical and physiological processes of microorganisms (Haferburg and Kothe, 2007). Yan et al. (2007) found that the adhesion ability of *V. alginolyticus* is influenced substantially by environmental factors, including temperature, pH, and salinity. Heavy metals, including Cu^2+^ and Pb^2+^, could also reduce *V. alginolyticus* adhesion (Kong et al., 2015).

The ocean is a complicated ecosystem with varied temperature, pH, salinity, and nutrients. The adhesion ability of *V. alginolyticus* has been shown to be affected by environmental factors. This could explain the seasonal vibriosis in the cultured large yellow croaker. However, the mechanisms governing *V. alginolyticus* adhesion under diverse environments are still unclear. Therefore, it is important to understand the mechanisms underlying the adhesion of *V. alginolyticus* and the effects of environmental factors on adhesion, which may aid in the development of preventive measures for reducing infection.

Previously, to further investigate the mechanism(s) of *V. alginolyticus* adhesion, we performed RNA-seq on *V. alginolyticus* treated with Cu^2+^, Pb^2+^, and low pH (Kong et al., 2015). These results showed that these stresses could significantly affect the TCA pathway.

The TCA pathway is a very important part of the central metabolic pathway, which supplies precursors for biosynthesis and is the source for energy in bacteria (Rezaei et al., 2015). An impaired TCA cycle was associated with a decrease in the virulence of pathogenic bacteria (Massilamany et al., 2011). However, few studies have reported the relationship between the TCA pathway, the adhesion of pathogenic bacteria and environmental factors (Stancik et al., 2002; Rajab et al., 2010; Gilbreath et al., 2012; Wallwiener et al., 2012; Michta et al., 2014; Vastano et al., 2014).

Therefore, in this study we attempted to: (1) determine the relationship between *V. alginolyticus* adhesion and the TCA pathway; (2) verify whether environmental factors affect *V. alginolyticus* adhesion through the TCA pathway.

## Materials and Methods

### Bacterial Strains and Growth Conditions

As previously described, *V. alginolyticus* (ND-01) was isolated from a naturally infected large yellow croaker and confirmed as pathogenic by artificial infection (Yan et al., 2001). The sample was stored at –80°C in physiological saline with 15% glycerol. *V. alginolyticus* was grown at 28°C either in Luria-Bertani (LB) broth or LB agar, which was supplemented with 2% NaCl. The bacteria were harvested by centrifugation at 4000 rpm for 15 min and re-suspended in phosphate-buffered saline (PBS, pH 7.4) after overnight incubation (Huang et al., 2015a).

To further validate the results of RNA-seq, the *V. alginolyticus* were stressed with Cu, Pb and low pH as previously mentioned (Kong et al., 2015), which can significantly reduce adhesion. The control group was cultured in LB broth (supplemented with 2% NaCl, pH = 7) (Kong et al., 2015). There were six replicates for each treatment. After overnight incubation at 28°C, the bacteria were harvested and used for RNA extraction and quantitative Real-Time PCR (qPCR).

To investigate the effect of different temperatures, *V. alginolyticus* was cultured in LB broth (supplemented with 2% NaCl, pH = 7) at 4, 15, 28, 37, and 44°C. There were six replicates for each treatment. Then, the bacteria were harvested and resuspended. The bacterial suspensions were equilibrated at the same temperature for 30 min. Then they were used to perform RNA extraction, qPCR, or an *in vitro* adhesion assay.

To assess the effects of changes in pH, *V. alginolyticus* was cultured in LB broth (supplemented with 2% NaCl) with different pH (pH = 5, 6, 7, 8, and 9). HCl and NaOH were used to adjust the pH. Bacterial cultures were washed with PBS (pH = 5, 6, 7, 8, and 9) (Yan et al., 2007) and adjusted to OD_560_≈0.3. Then, RNA extraction, qPCR, and *in vitro* adhesion assay were performed. There were six replicates for each treatment.

To evaluate the influence of different salinities, *V. alginolyticus* was cultured in LB broth with different salinities (0.8, 1.5, 2.5, 3.5, and 4.5%). There were six replicates for each treatment. Bacterial cultures were washed by PBS with different salinities (0.8, 1.5, 2.5, 3.5, and 4.5%) (Yan et al., 2007) and adjusted to OD_560_≈0.3. Then, the bacterial suspensions were used to perform an *in vitro* adhesion assay, RNA extraction, and qPCR.

To evaluate the influence of starvation, *V. alginolyticus* was suspended in normal PBS. The bacterial suspensions were adjusted to OD_560_≈0.3 and kept at starvation at 28°C for 1, 3, 5, and 7 days, respectively (Yan et al., 2010) before being sampled for the *in vitro* adhesion assay, RNA extraction, and qPCR. There were six replicates for each treatment. At the same time, the viable cells of *V. alginolyticus* were counted by plate count (PC) (Yi et al., 2008).

### Gene Ontology (GO) and KEGG Pathways Annotation for Differential Expression Genes (DEGs)

In our previous research (Kong et al., 2015), we presented the first RNA-seq data from *V. alginolyticus* cultured under stress conditions, including Cu, Pb, low pH, as well as normal conditions. The data were deposited in the NCBI Sequence Read Archive (SRA) under the accession number SRP049226.

We performed GO annotation of the unigenes with the Blast2GO program. Then, WEGO software was used to carry out GO functional classification. The calculated *P* value went through Bonferroni Correction, taking a corrected *P*-value ≤ 0.05 as a threshold for significance. GO terms fulfilling this condition were defined as significantly enriched GO terms in DEGs.

The KEGG pathway annotation was carried out using Blastall software against the KEGG (http://www.genome.jp/kegg/) database. The *Q* value was defined to be the FDR analog of the *P*-value. Pathways with *Q*-value ≤ 0.05 were regarded as significantly enriched in DEGs.

### Preparation of Mucus

Healthy large yellow croakers caught by commercial fishermen from marine culture cages in the city of Ningde in the Fujian province of China were used for mucus preparation in accordance with our previous method (Huang et al., 2015b). After washing with sterile PBS (0.01 mol/L, pH 7.2), the skin mucus was harvested by scraping the surface of the skin with a plastic spatula to remove the mucus gel layer. This layer was then homogenized in PBS. The mucus preparations were centrifuged twice (20,000 *g*, 4°C, 30 min) to remove particulate materials and then filtered through 0.45 and 0.22 μm filters. The mucus samples were adjusted to 1 mg protein/mL PBS using the Bradford’s method (Bradford, 1976).

### Total RNA Extraction and Reverse Transcription

Total RNA of the bacteria was extracted with Trizol (Invitrogen, Carlsbad, CA, USA) as previously described (Kong et al., 2015). First-strand cDNA was synthesized with a Reverse Transcription kit (Dongsheng Biotech, China) according to the manufacturer’s protocol.

### Transient Gene Silencing

In our previous research (Wang et al., 2015), we showed that using RNA-seq along with RNAi is a highly accurate and efficient way to describe functional genes. Therefore, we chose the same strategy in the present study. Short interfering RNA (siRNA) was synthesized by GenePharma Co. Ltd. (Shanghai, China) according to the gene sequences. Negative control siRNA and treatment siRNA sequences are listed in **Table 1**.

**Table 1:** siRNA sequences.

Target gene	siRNA sequences
*pckA*	F:5’ GCUACGACAGAAGAGCAUATT 3’
	R:5’ UAUGCUCUUCUGUCGUAGCTT 3’
*pdhB*	F: 5’ GCACACUGCUGGCUUUAAATT 3’
	R:5’ UUUAAAGCCAGCAGUGUGCTT 3’
*acnA*	F: 5’ GCUUUGAACCUGAUGCUUUTT 3’
	R: 5’ AAAGCAUCAGGUUCAAAGCTT 3’
*sdhC*	F: 5’ GGAUCUAGGUCACUUUGAATT 3’
	R: 5’ UUCAAAGUGACCUAGAUCCTT 3’
*sucC*	F: 5’ GCUCUUUACCGUCAGCCUATT 3’
	R: 5’ UAGGCUGACGGUAAAGAGCTT 3’
*oorA*	F: 5’ CCUUGAGCUUGCUGGCUAUTT 3’
	R: 5’ AUAGCCAGCAAGCUCAAGGTT 3’
Negative control	F: 5’ UUCUCCGAACGUGUCACGUTT 3’
	R: 5’ ACGUGACACGUUCGGAGAATT 3’


Electro-transformation of *V. alginolyticus* strains was carried out using a Bio-Rad MicroPulser (Bio-Rad Laboratories, Inc.) in accordance with our previous study (Huang et al., 2015a). After electroporation, 900 μl of LB medium was added immediately and then incubated at 28°C for 1, 3, 6, 9, and 12 h prior to RNA extraction, RT-PCR and bacterial adhesion assay.

### Quantitative Real-Time PCR Assay

The expression levels of differentially expressed genes (DEGs) in the TCA pathway identified via transcriptome sequencing were verified using qPCR with SYBR Green qPCR Mix (Dongsheng Biotech, China) according to the manufacturer’s instructions. The expression levels were normalized with 16S RNA, which was calculated using the 2^-ΔΔCt^ method (*n* = 6). We designed the primers listed in **Table 2** according to the guidelines published by Thornton and Basu (2011).

**Table 2:** Primers used in quantitative real-time PCR (qPCR).

Primer	Sequence
pckA-for	5’ ATGAGCACGGTTGGGATG 3’
pckA-rev	5’ GATACTGGCTTAACGATGTTGTC 3’
pdhB-for	5’ GTCACTTCAAGCCGCACAG 3’
pdhB-rev	5’ CTCAGAGCCCACACCACAAG 3’
acnA-for	5’ CGACCTTTGCTAACCC 3’
acnA-rev	5’ GCCTTGACCGTAATCC 3’
sdhC-for	5’ CTGACATCGTCGATAGCTTC 3’
sdhC-rev	5’ GCCAATAATGATAATACTGCTG 3’
sucC-for	5’ CTTTATGGGTCTTGGCACTATG 3’
sucC-rev	5’ GTTCCCACTGAGCTGCGTG 3’
oorA-for	5’ CGAAACAAGGAAACGATG 3’
oorA-rev	5’ CACCAAACCGCCTTCAAC 3’
16S-for	5’ GGGGAGTACGGTCGCAAGAT 3’
16S-rev	5’ CGCTGGCAAACAAGGATAAGG 3’


### *In Vitro* Adhesion Assay

Adhesion ability was determined in accordance with our published method (Kong et al., 2015). A total of 50 μL of mucus was evenly spread on a 22 mm × 22 mm glass slide and fixed with methanol for 20 min. Then, 1 ml of bacterial suspension (10^8^ CFU/ml) was placed on the mucus-coated glass slides, incubated for 2 h at 28°C in a humidified chamber, and washed with PBS five times. Finally, the bacteria were fixed using 4% methanol for 30 min, stained with crystal violet for 3 min, and counted under a microscope (× 1,000). Each group was conducted in five trials, and 20 fields of view were selected. Two kinds of negative control were performed: (1) using PBS instead of bacterial suspension and (2) using PBS instead of mucus.

### Data Processing

All data from this study were expressed as the mean ± standard deviation and statistically analyzed with SPSS18.0. The difference between the mean values was determined by one-way ANOVA followed by Dunnett’s multiple comparison tests. A value of *P* < 0.05 was used to indicate a significant difference.

REST2008 (Pfaffl et al., 2002) was used to calculate the relative expression of mRNA target genes in qPCR as described before (Kong et al., 2015). Significant differences between groups were determined by ANOVA followed by Tukey’s LSD.

## Results

### RNA-seq Screening for DEGs

In our previous research (Kong et al., 2015), we showed that low pH reduced adhesion by 56.58%, while Cu and Pb reduced adhesion by 37.41 and 39.26%, respectively. Therefore, we performed RNA-seq on *V. alginolyticus* stressed with Cu, Pb, and low pH. The data were deposited in the NCBI SRA under the accession number SRP049226.

RNA-seq and DEGs analysis yielded 1,637, 1,085, and 1,791 DEGs in the Cu-, Pb-, and low pH-treated groups compared with the control group, respectively. GO analysis indicated that the functional distribution of the DEGs from different stressed groups was similar. Most of the biological process genes were involved in cellular processes, metabolic processes, establishment of localization, and localization. Most of the cellular component genes encoded proteins associated with cells, cell parts, membranes and membrane parts. Most of the molecular function genes were associated with binding and catalytic activity. KEGG analysis yielded 164 KEGG pathways, for example: ‘The TCA Pathway’.

The TCA pathway was selected for further research. DEGs in the TCA pathway varied across stressed conditions (**Supplementary Images 1**–**3**). There were six commonly down-regulated DEGs: phosphoenolpyruvate carboxykinase ATP (*pckA*), pyruvate dehydrogenase E1 component beta subunit (*pdhB*), aconitate hydratase (*acnA*), succinate dehydrogenase (*sdhC*), succinyl-CoA synthetase beta subunit (*sucC*), and 2-oxoglutarate ferredoxin oxidoreductase subunit alpha (*oorA*). These genes were significantly changed in all stress groups. Thus, they appeared to be the most sensitive to environmental stresses.

### Validation of the Results of RNA-seq

**FIGURE 1:**
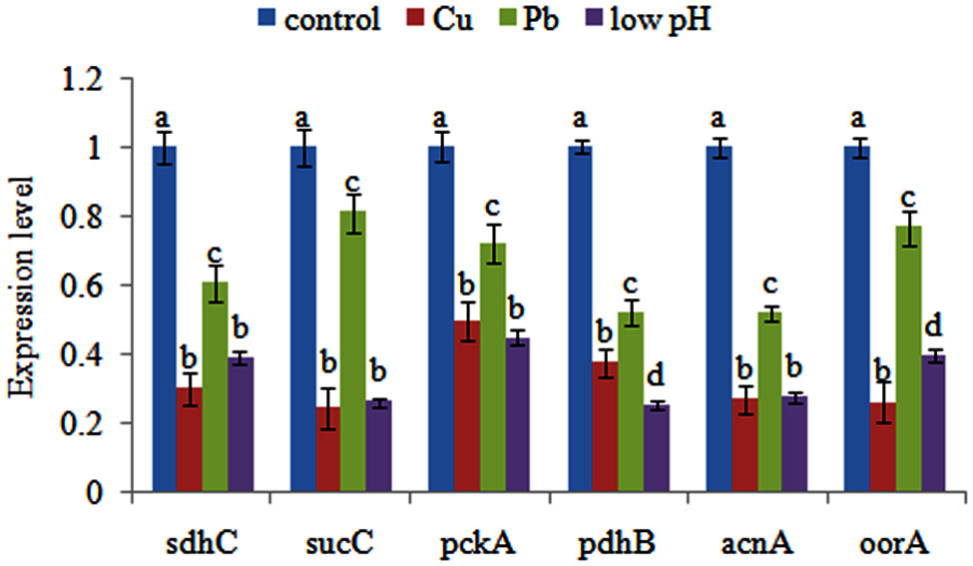
**Quantitative real-time PCR (qPCR) analysis of the expression of *sdhC*, *sucC*, *pckA*, *pdhB*, *acnA*, and *oorA* after stress treatments in comparison to untreated control.** The data are presented as the means ± SD, *n* = 6. The means of the treatments not sharing a common letter are significantly different at *P* < 0.05.

qPCR was performed on these six genes to validate the RNA-seq results. Our qPCR results matched our sequencing results: the Cu, Pb, and low pH treatments significantly down-regulated the expression of *sdhc* (by 3.35-, 1.65-, and 2.58-fold, respectively), *sucC* (by 4.12-, 1.24-, and 3.85-fold, respectively), *pckA* (by 2.02-, 1.39-, and 2.24-fold, respectively), *pdhB* (by 2.68-, 1.92-, and 3.99-fold, respectively), *acnA* (by 3.71-, 1.93-, and 3.64-fold, respectively) and *oorA* (by 3.86-, 1.30-, and 2.52-fold, respectively) (**Figure 1**). These reinforced the reliability of the sequencing data.

### Effects of Transient Gene Silencing

**FIGURE 2:**
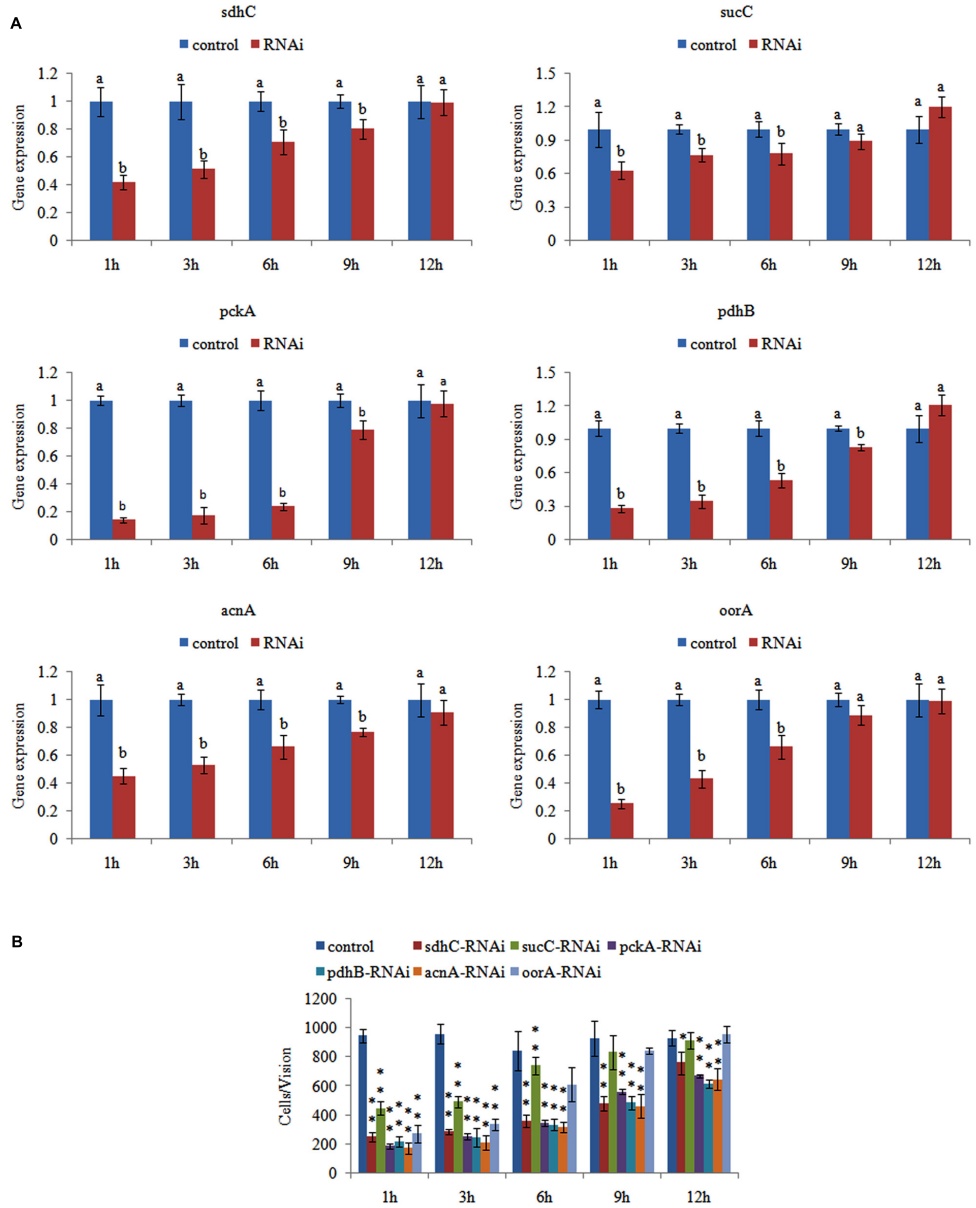
**Transient RNAi reduced the adhesion of *Vibrio alginolyticus*.** **(A)** QPCR analysis of the expression of *sdhC*, *sucC*, *pckA*, *pdhB*, *acnA*, and *oorA* after transient gene silencing at 1, 3, 6, 9, and 12 h in comparison to the control. The data are presented as the means ± SD, *n* = 6. The means of the treatments not sharing a common letter are significantly different at *P* < 0.05. **(B)** The adhesion capacity to mucus of transient silenced *V. alginolyticus* at 1, 3, 6, 9, and 12 h. The data are presented as the means ± SD, *n* = 3. ^∗∗^*P* < 0.01 versus the control group. ^∗^*P* < 0.05 versus the control group.

After RNAi, the gene expression levels were detected at 1, 3, 6, 9, and 12 h. The gene expression levels after RNAi were normalized against the corresponding control (scrambled) siRNA treatments. The expression of these genes decreased significantly at 1–6 h. After 6 h, some of the genes (*sucC* and *oorA*) were not significantly changed compared with control groups, while the other genes (including *pckA*, *pdhB*, *acnA*, and *sdhC*) were still significantly changed until 12 h (**Figure 2A**). The reduction in the target genes indicated that the siRNAs functioned properly.

We then determined the adhesion ability of *V. alginolyticus* after RNAi. The *in vitro* adhesion assay showed that RNAi led to a significant reduction of adhesion (**Figure 2B**), while the reduction of adhesion was alleviated over time. The trend of qPCR and *in vitro* adhesion assay results after RNAi was quite similar. These results indicated that RNAi significantly impaired the adhesion ability of *V. alginolyticus*.

Interestingly, siRNA treatment also significantly down-regulated the expression of *sdhc* (by 2.39-fold), *sucC* (by 1.60-fold), *pckA* (by 7.18-fold), *pdhB* (by 3.59-fold), *acnA* (by 2.22-fold), and *oorA* (by 4.01-fold) at 1 h, while the adhesion was significantly reduced by 3.82-, 2.13-, 5.18-, 4.35-, 5.51-, and 3.50-fold, respectively. Therefore, *acnA* appeared to be the gene most closely related to adhesion among these genes.

### Effect of Different Temperatures

**FIGURE 3:**
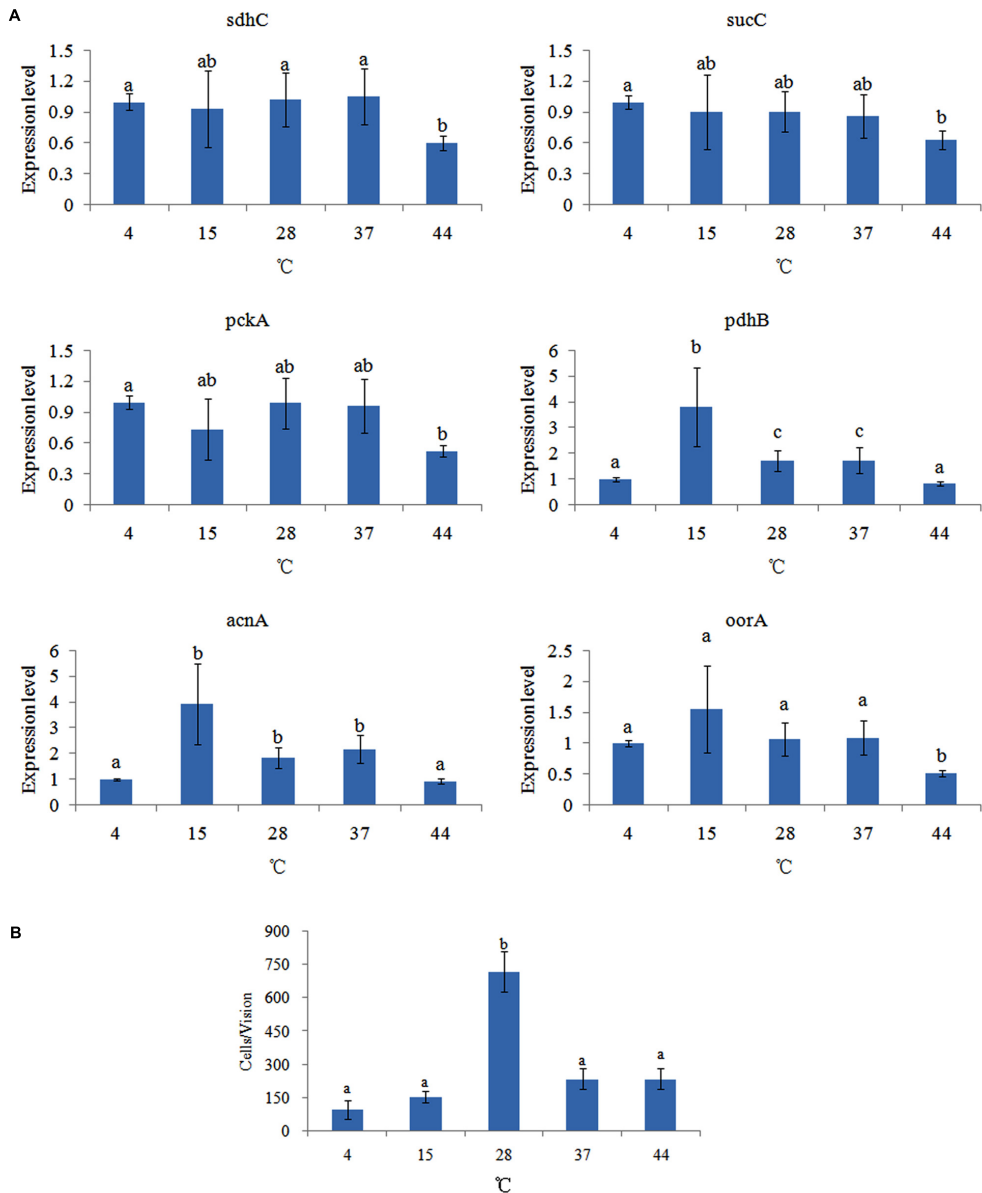
**Temperature affected the adhesion of *V. alginolyticus*.** **(A)** qPCR analysis of the expression of *sdhC*, *sucC*, *pckA*, *pdhB*, *acnA*, and *oorA* after treated with different temperatures. The data are presented as the means ± SD, *n* = 6. The means of the treatments not sharing a common letter are significantly different at *P* < 0.05. **(B)** The adhesion capacity to mucus of *V. alginolyticus* cultured under different temperatures. The data are presented as the means ± SD, *n* = 3. The means of the treatments not sharing a common letter are significantly different at *P* < 0.05, as assessed using one-way ANOVA followed by Dunnett’s test.

The effect of different temperatures on the expression of the genes was compared (**Figure 3A**). Temperatures affected the expression of different genes in different ways. Among these genes, the expression of *pckA*, *sdhC*, *sucC*, and *oorA* had no significant difference at 4, 15, 28, and 37°C, which indicated that temperature had little influence on the expression of these genes. However, these genes had their lowest expression levels at 44°C. Interestingly, the expression of *pdhB* and *acnA* were significantly changed at 4, 15, 28, 37, and 44°C, with their highest expression levels at 15°C.

The effect of different temperatures on bacterial adhesion was also detected (**Figure 3B**). The change of adhesion ability under different temperatures followed an inverted U-shaped trend. The number of *V. alginolyticus* adhered to the skin mucus of large yellow croaker at 28°C was significantly higher than at the other temperatures, which was consistent with previous reports (Toren et al., 1998; Yan et al., 2007). This might explain why *V. alginolyticus* infection occurs more frequently in early summer.

The *in vitro* adhesion assay and qPCR showed different trends under different temperatures. This indicated that temperature could affect *V. alginolyticus* adhesion, but that the TCA pathway might be not involved in the regulatory network governing adhesion in response to different temperatures.

### Effects of Different pH Levels

**FIGURE 4:**
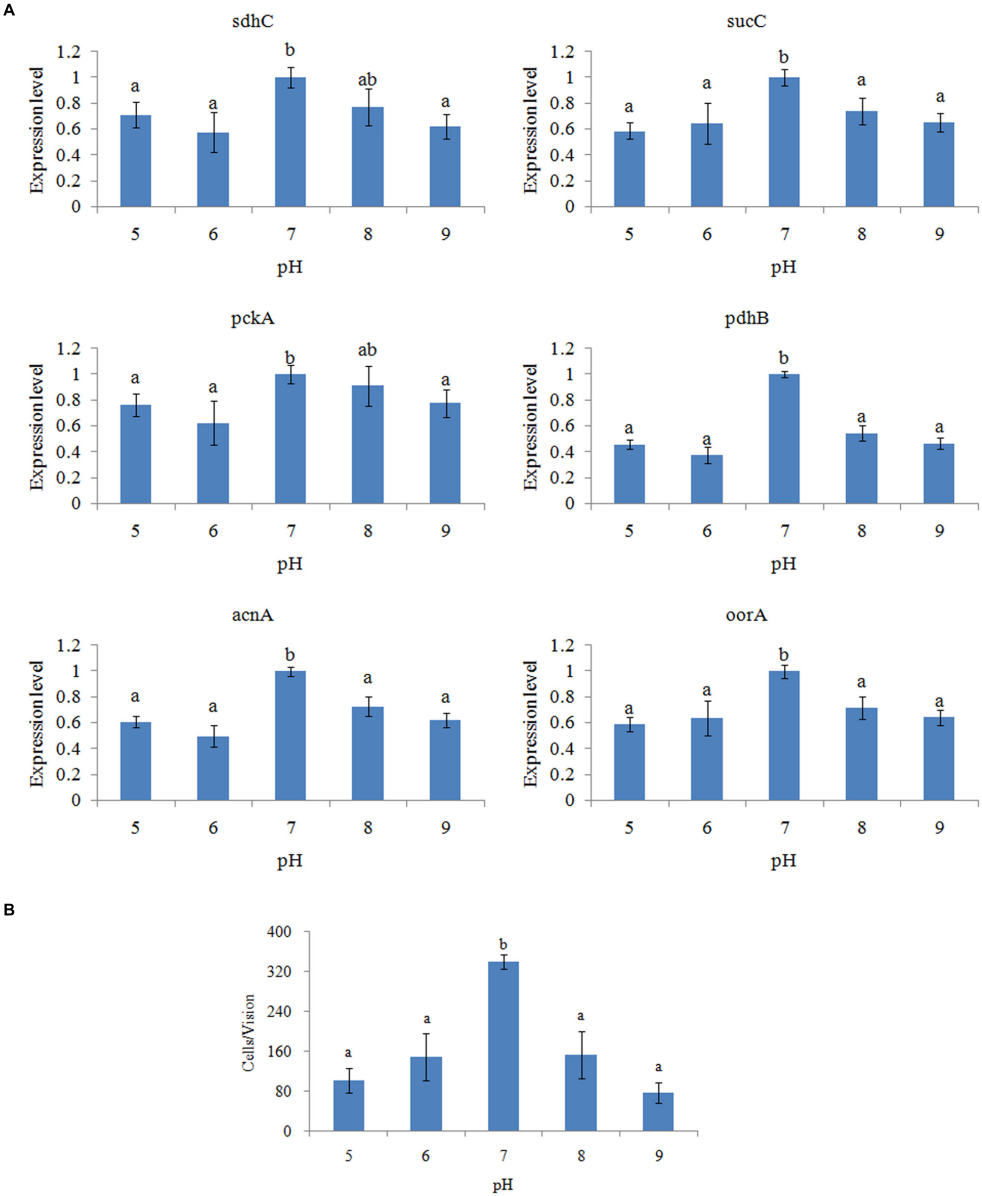
**pH affected the adhesion of *V. alginolyticus*.** **(A)** qPCR analysis of the expression of *sdhC*, *sucC*, *pckA*, *pdhB*, *acnA*, and *oorA* after treated with different pH. The data are presented as the means ± SD, *n* = 6. The means of the treatments not sharing a common letter are significantly different at *P* < 0.05. **(B)** The adhesion capacity to mucus of *V. alginolyticus* cultured under different pH. The data are presented as the means ± SD, *n* = 3. The means of the treatments not sharing a common letter are significantly different at *P* < 0.05, as assessed using one-way ANOVA followed by Dunnett’s test.

The expression of genes in *V. alginolyticus* cultured at different pH levels was detected (**Figure 4A**), which displayed similar inverted U-shaped trend. The highest expression levels were observed at pH 7.0. The *oorA* gene appeared to be the most sensitive to different pH, while *acnA* appeared to be the least sensitive.

The adhesion ability of *V. alginolyticus* to skin mucus at different pH levels was also measured (**Figure 4B**). The adhesion ability of *V. alginolyticus* at different pH levels also displayed an inverted U-shaped trend and reached the peak at pH 7.0, which was consistent with previous reports (Varma et al., 2010).

The trends of qPCR and the *in vitro* adhesion assays under different pH were quite similar. This indicated that pH can affect adhesion of *V. alginolyticus* and that the TCA pathway might be involved in the regulatory network governing adhesion under different pH.

Furthermore, it seemed that the difference between the expression level of *pdhB* at pH 7.0 and other conditions was larger than other genes. Therefore, *pdhB* appeared to be the most sensitive to pH among the genes tested.

### Effects of Different Salinity Treatments

**FIGURE 5:**
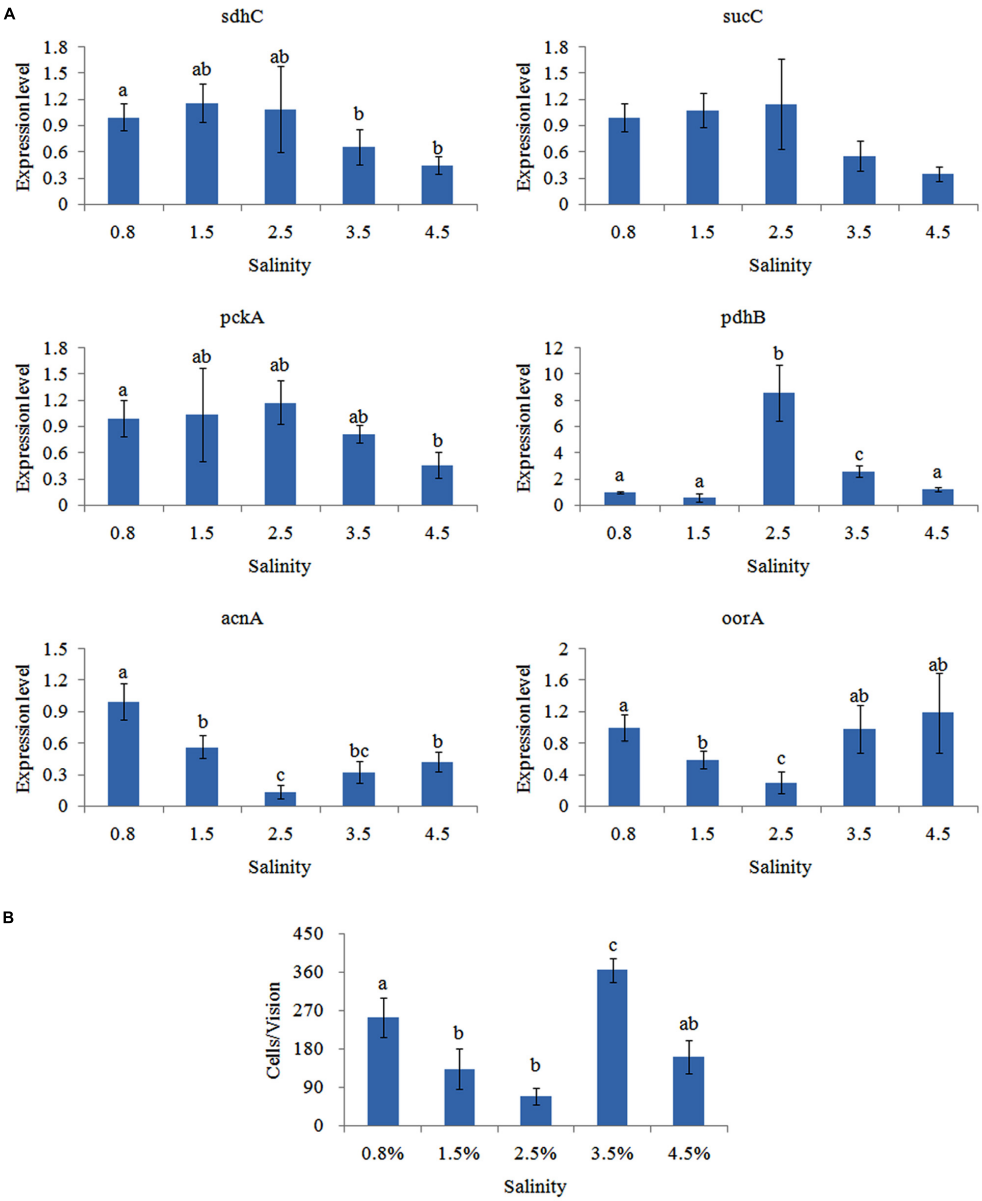
**Salinity affected the adhesion of *V. alginolyticus*.** **(A)** qPCR analysis of the expression of *sdhC*, *sucC*, *pckA*, *pdhB*, *acnA*, and *oorA* after treated with different salinities. The data are presented as the means ± SD, *n* = 6. The means of the treatments not sharing a common letter are significantly different at *P* < 0.05. **(B)** The adhesion capacity to mucus of *V. alginolyticus* cultured under different salinities. The data are presented as the means ± SD, *n* = 3. The means of the treatments not sharing a common letter are significantly different at *P* < 0.05, as assessed using one-way ANOVA followed by Dunnett’s test.

The effects of salinity on gene expression were quite different. The expression of *pckA*, *sdhC*, and *sucC* was not significantly changed at the 0.8, 1.5, 2.5, and 3.5% salinity, while their expression was noticeably down-regulated at 4.5% salinity (**Figure 5A**). The expression of *pdhB* displayed an inverted U-shaped trend and reached the highest expression level at the 2.5% salinity. Inversely, the expression of *acnA* and *oorA* displayed a U-shaped trend and reached the lowest expression level at the 2.5% salinity.

Adhesion of *V. alginolyticus* at different salinities was detected (**Figure 5B**). Maximum adhesion was achieved at the 3.5% salinity. Interestingly, adhesion was significantly higher at.8% salinity compared to 1.5, 2.5, or 4.5% salinity. This was consistent with previous reports (Yan et al., 2007).

Whereas, the trend of qPCR and *in vitro* adhesion assay at different salinities was quite different except for *oorA*. This indicated that salinity can affect adhesion of *V. alginolyticus* and that *oorA* might be involved in the regulatory network governing adhesion in response to different salinities.

### Effects of Starvation

**FIGURE 6:**
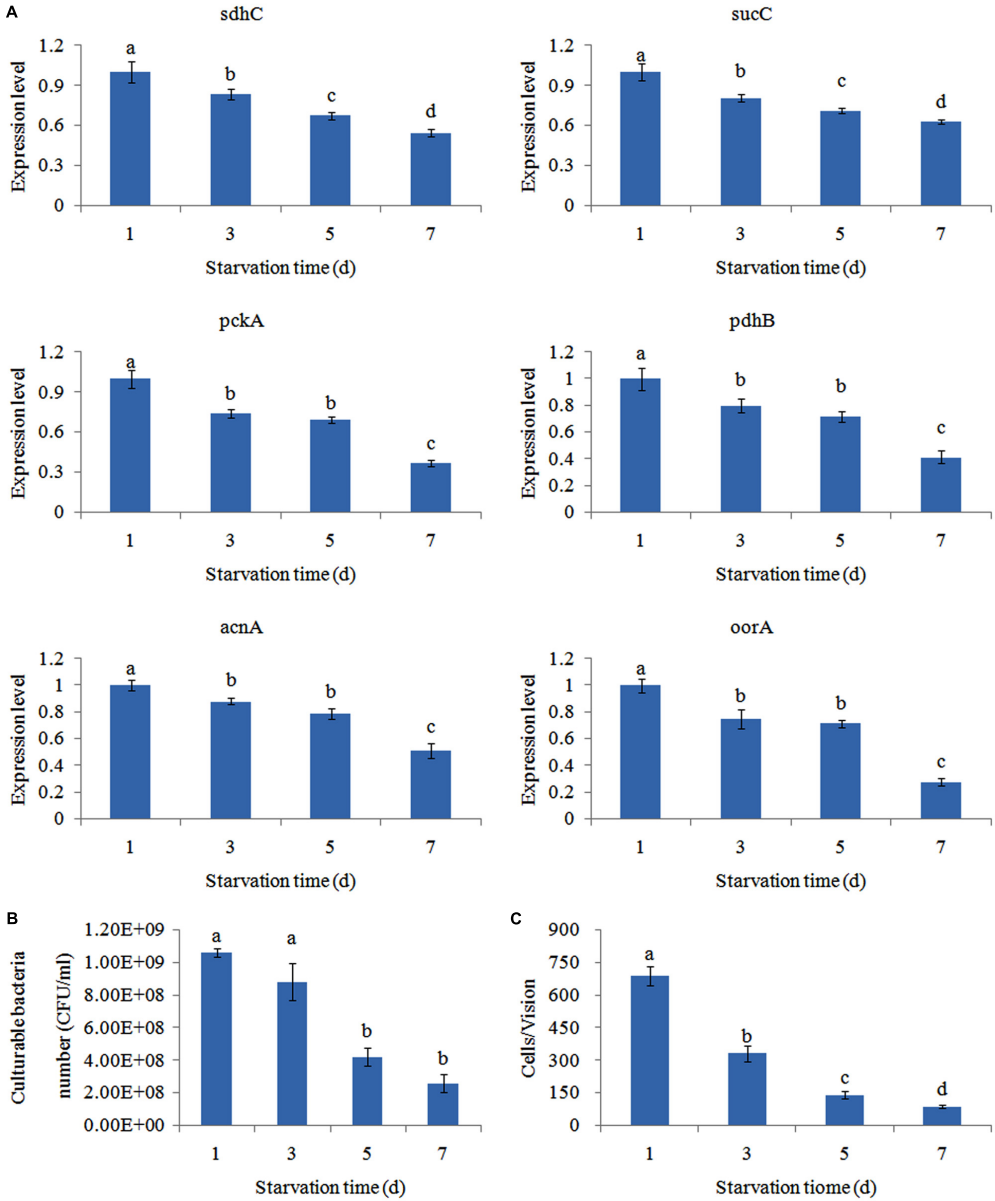
**Starvation affected the adhesion of *V. alginolyticus*.** **(A)** qPCR analysis of the expression of *sdhC*, *sucC*, *pckA*, *pdhB*, *acnA*, and *oorA* after starvation for 1, 3, 5, and 7 days. The data are presented as the means ± SD, *n* = 6. The means of the treatments not sharing a common letter are significantly different at *P* < 0.05. **(B)** Count of culturable bacteria after starvation for 1, 3, 5, and 7 days. The data are presented as the means ± SD, *n* = 6. The means of the treatments not sharing a common letter are significantly different at *P* < 0.05. **(C)** The adhesion capacity to mucus of *V. alginolyticus* after starvation for 1, 3, 5, and 7 days. The data are presented as the means ± SD, *n* = 3. The means of the treatments not sharing a common letter are significantly different at *P* < 0.05, as assessed using one-way ANOVA followed by Dunnett’s test.

The expression of the genes was significantly reduced in a time-dependent manner after starvation (**Figure 6A**). This showed that the number of viable bacteria was not significantly changed before 3 days of starvation. Bacterial adhesion to skin mucus was substantially reduced the longer *V. alginolyticus* cultures were starved (**Figure 6B,C**). Therefore, the decline of the number of bacteria adhered to skin mucus was mainly due to the decline of the bacterial adhesion ability rather than the decline of the number of bacteria in the suspension. This suggested that vibriosis caused by *V. alginolyticus* was more likely to occur in eutrophic seawater rather than oligotrophic seawater. Since the trends of gene expression and adhesion under starvation was quite similar, starvation might affect the adhesion of *V. alginolyticus* through perturbing the TCA pathway. It is reported that starvation inhibits continuous bacterial protein synthesis via the TCA pathway, which is required for stable bacterial adherence (Wang and Leung, 2000). This might explain the reduced adhesive capability of starved *V. alginolyticus* (Yan et al., 2007).

In addition, starvation significantly down-regulated the expression of *sdhc* (by 1.20-fold), *sucC* (by 1.24-fold), *pckA* (by 1.35-fold), *pdhB* (by 1.25-fold), *acnA* (by 1.14-fold), and *oorA* (by 1.33-fold) at 3 day, respectively. Therefore, *pckA* appeared to be the most sensitive gene to starvation among these genes.

## Discussion

Bacterial adhesion is a very complex process. It involves not only adherence to specific receptors within the mucus or epithelial cell surface receptors but also the bacterial attraction to the mucosal surface. This means that bacteria sense and respond to a chemical stimulus (chemotaxis), flagella assembly, energy production, and biosynthesis; and other biological processes are involved in the regulation of adhesion. At the same time, these biological processes could affect each other. For example, energy production can affect chemotaxis and flagella assembly, changes in biosynthesis can affect the assembly of functional flagella. These biological processes are tightly regulated by protein coding genes and ncRNAs. Some of these processes could be perturbed by environmental factors, which can lead to changes in adhesion ability.

In our previous study (Huang et al., 2015a; Kong et al., 2015), we presented the first RNA-seq data from *V. alginolyticus* cultured under stress conditions (including Cu, Pb, and low pH) that reduced adhesion. Based on our RNA-seq and bio-informatics analysis, we found pathways (including the flagellar assembly pathway and the TCA pathway) and ncRNAs that might be closely related to adhesion (Huang et al., 2015a; Kong et al., 2015; Wang et al., 2015). We have proved that perturbation of the flagellar assembly pathway (Wang et al., 2015) and three ncRNAs (Huang et al., 2015a) are associated with a decrease in adhesion ability. In the present study, we investigated the relationship between the TCA pathway and adhesion.

According to the results of our RNA-seq analysis, there were six commonly down-regulated DEGs: *pckA*, *pdhB*, *acnA*, *sdhC*, *sucC*, and *oorA* in the TCA pathway. These genes were significantly different in all stressed groups and thus may be the genes most sensitive to environmental stressors. Previous studies have shown that *acnA*, *sucC*, and *oorA* are sensitive to stress conditions (Gruer and Guest, 1994; Cunningham et al., 1997; Stancik et al., 2002; Ojima et al., 2008; Alfreider and Vogt, 2012; Gilbreath et al., 2012; Michta et al., 2014), which supports our hypothesis.

We examined the relationship between *pckA*, *pdhB*, *acnA*, *sdhC*, *sucC*, and *oorA* and adhesion via qPCR, RNAi, and an adhesion assay. Our results showed that the adhesion ability of *V. alginolyticus* after RNAi was significantly impaired. This indicates that *pckA*, *pdhB*, *acnA*, *sdhC*, *sucC*, and *oorA* are closely related to adhesion, which supported the results of RNA-seq and our hypothesis.

Mutations in *pckA* have profound biological effects on a variety of bacterial species. Although no direct evidence has indicated the existence of a relationship between *pckA* and adhesion, it has been shown that deletion of the *pckA* gene leads to a reduction in the bacterial infection ability (Liu et al., 2005), while adhesion is a critical step in the initial stage of infection (Thune et al., 1993). *pdhB* is a pivotal metabolic gene, and the *pdhB* deletion mutant displayed less efficient adhesion than the wild type strain to FN (Rajab et al., 2010; Wallwiener et al., 2012; Vastano et al., 2014). *acnA* is an essential component of the TCA cycle (Gruer et al., 1997; Baothman et al., 2013). Research on *Salmonella enterica* has shown that *acnA* might be involved in the regulation of flagellar assembly and adhesion to the surface of J774 macrophage-like cells (Tang et al., 2004). *sdhC* is known to contribute to the pathogenicity of bacteria (Nakamura et al., 1996; Mercado-Lubo et al., 2008; Dahal et al., 2013), but its role in infection has yet to be elucidated. Our research indicates that *sdhC* might affect virulence through regulation of adhesion ability. *sucC* and *oorA* are both essential components of the TCA cycle, which supplies precursors for biosynthesis and provides energy (Buck et al., 1986; Gilbreath et al., 2012). Since no previous research correlated them with the virulence of bacteria, this was the first report to discuss the relationship between them and the virulence of bacteria. We speculated that *sucC* and *oorA* might affect adhesion by perturbing the supply of precursors for biosynthesis and the energy supply.

We examined the relationships between these genes, adhesion and varied environmental stresses including temperature, pH, salinity and starvation with qPCR and an adhesion assay. According to our results, adhesion of *V. alginolyticus* is influenced substantially by environmental factors. The six genes we examined exhibited different responses to different environmental stresses. In addition, the same environmental stress leads to different changes in expression in different genes. This indicates that the TCA pathway plays a key role in the adhesion process of *V. alginolyticus* and is sensitive to some environmental stresses, especially pH and starvation. Since the pH in seawater is relatively stable, the availability of nutrients might be the chief factor affecting the TCA pathway thus affecting adhesion.

Interestingly, our previous research (Huang et al., 2015a; Wang et al., 2015) showed that Hg exposure could significantly perturb the flagellar assembly pathway and the 3 previously mentioned ncRNAs in a similar manner to Cu, Pb, and low pH. However, Hg exposure could not affect the TCA pathway. This indicates that the down-regulation of genes in the TCA is a result of a direct effect of environmental stressors instead of a sub-effect of the ncRNAs (Huang et al., 2015a) or flagellar assembly (Wang et al., 2015). Simultaneously, because the TCA pathway provided the materials and energy needed for gene expression, ncRNA regulation and flagellar assembly, the TCA pathway might affect adhesion both directly and indirectly.

Furthermore, because the TCA pathway is sensitive to environmental factors, it might be a potent target for therapeutic intervention in seasonal bacterial disease. Further research is still necessary.

## Author Contributions

LH and LiH participated in the microbiology studies, carried out the analysis, and interpretation of data immunoassays, participated in the sequence alignment and drafted the manuscript. QY conceived of the study, participated in its design and coordination, and participated in the sequence alignment and drafted the manuscript. YQ participated in the design of the study and performed the statistical analysis. YM participated in the design of the study and performed the statistical analysis. ML participated in the microbiology studies. XX participated in the design of the study. JZ participated in the microbiology studies.

## Conflict of Interest Statement

The authors declare that the research was conducted in the absence of any commercial or financial relationships that could be construed as a potential conflict of interest.

## Supplementary Material

**IMAGE 1 | The overall scheme for genes involved in the TCA pathway that were significantly affected by Cu stress.** Green and red indicate decreased and increased expression, respectively.

**IMAGE 2 | The overall scheme for genes involved in the TCA pathway that were significantly affected by Pb stress.** Green and red indicate decreased and increased expression, respectively.

**IMAGE 3 | The overall scheme for genes involved in the TCA pathway that were significantly affected by low pH stress.** Green and red indicate decreased and increased expression, respectively.
