# Supplementary figures and images for: The TCA Pathway is an Important Player in the Regulatory Network Governing Vibrio alginolyticus Adhesion Under Adversity
Source: Front Microbiol. 2016 Feb 2;7:40. doi: 10.3389/fmicb.2016.00040 (PMC4735382; doi:10.3389/fmicb.2016.00040)

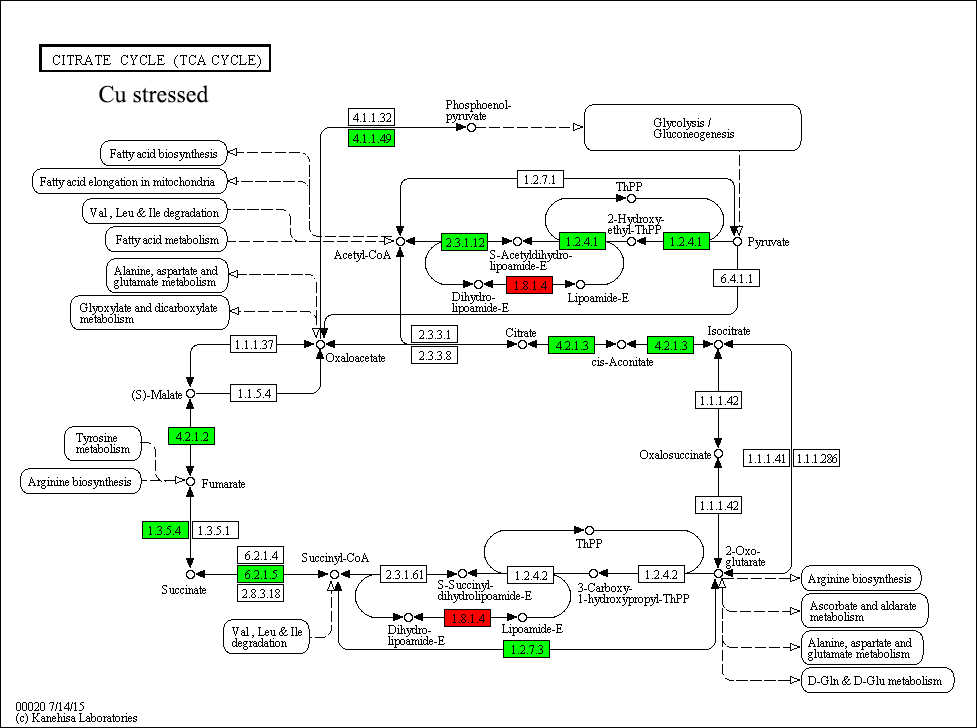

Supplement: Supplementary file 1 [file Image_1.TIF]

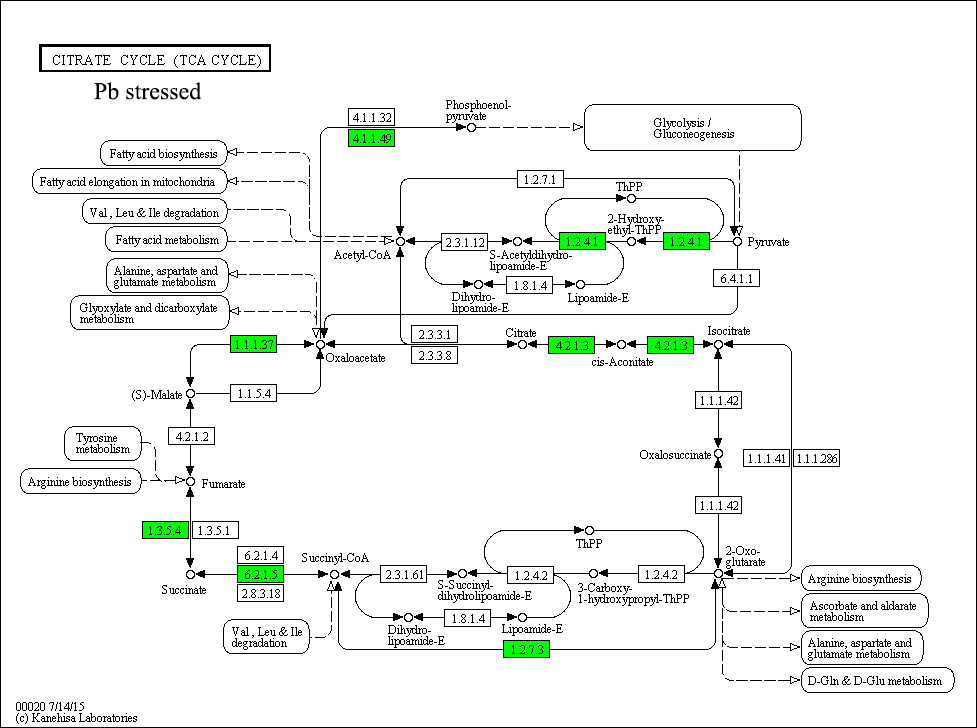

Supplement: Supplementary file 2 [file Image_2.TIF]

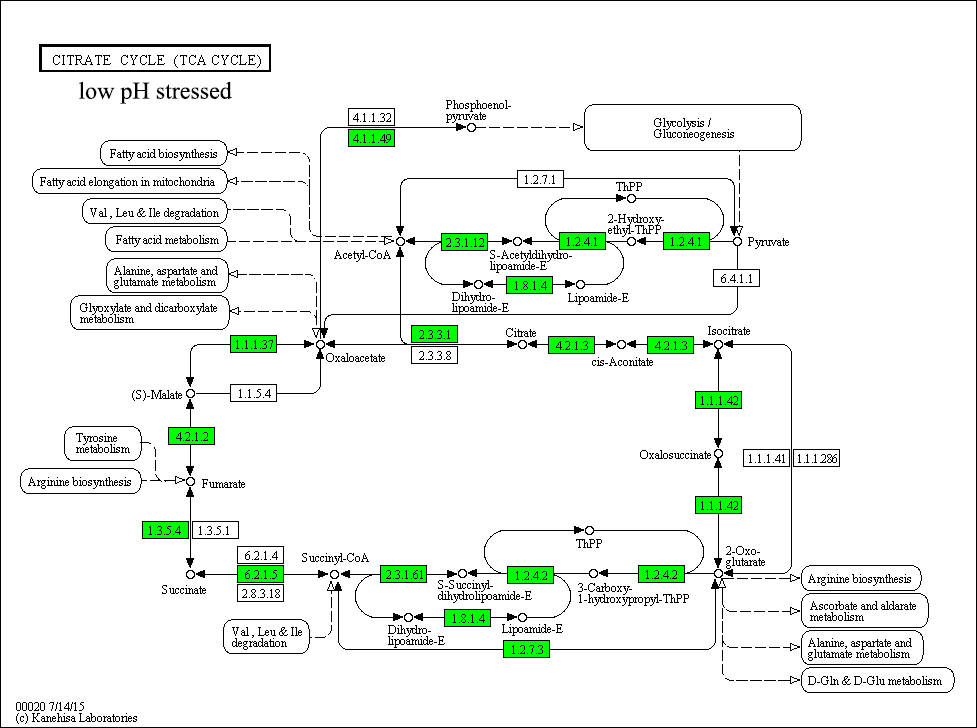

Supplement: Supplementary file 3 [file Image_3.TIF]
